# Factors Affecting Recruitment into Psychiatry: a Canadian Experience

**DOI:** 10.1007/s40596-014-0269-6

**Published:** 2015-01-13

**Authors:** Timothy Lau, Delara Zamani, Elliott Kyung Lee, Khashayar D. Asli, Jasbir Gill, Nancy Brager, Raed Hawa, Wei-Yi Song, Eunice Gill, Renee Fitzpatrick, Natasja M. Menezes, Vu H. Pham, Alan Bruce Douglass, Suzanne Allain, Greg B. Meterissian, Nadine Gagnon, Hadi Toeg, Cheryl Murphy

**Affiliations:** 1 University of Ottawa, Ottawa, ON Canada; 2 University of Sherbrooke, Sherbrooke, QC Canada; 3 Memorial University of Newfoundland, St John’s, NL Canada; 4 University of Calgary, Calgary, AB Canada; 5 University of Toronto, Toronto, ON Canada; 6 University of British Columbia, Vancouver, BC Canada; 7 University of Manitoba, Winnipeg, MB Canada; 8 Queens University, Kingston, ON Canada; 9 McMaster University, Hamilton, ON Canada; 10 University of Saskatchewan, Saskatoon, SK Canada; 11 Northern Ontario School of Medicine, Greater Sudbury, ON Canada; 12 McGill University, Montréal, QC Canada; 13 Laval University, Quebec, QC Canada; 14 Dalhousie University, Halifax, NS Canada

**Keywords:** Psychiatry, Career choice, Recruitment, Medical students, Pre-clerkship

## Abstract

**Objective:**

There is a projected shortage of psychiatrists in Canada in forthcoming years. This study assessed factors in medical school education that are associated with students selecting psychiatry first and matching as a discipline.

**Method:**

The Canadian Organization of Undergraduate Psychiatry Educators (COUPE) conducted telephone interviews and sent e-mail questionnaires to the 17 medical schools across Canada; all schools provided data for 2012. Relevant data were obtained from the Canadian Resident Matching Service. Statistics were performed using v12 STATA program, and significance was set at a *p* value of <0.05.

**Results:**

Medical student enrollment ranged from 54 to 266 students (mean = 158 ± 16). Of these students, 4.9 ± 0.6 % ranked psychiatry as their first choice for residency. Final match results yielded similar numbers at 5.0 ± 0.6 %. Ten out of 17 programs filled all psychiatry residency positions, whereas the remaining 7 programs had vacancy rates from 5 to 100 % (mean = 43.4 ± 15.1 %). Medical students were exposed to an average of 2.8 ± 0.5 pre-clerkship psychiatry weeks and 6.2 ± 0.3 clerkship weeks. Linear regression analysis demonstrated that the percentage of graduating medical students entering a psychiatry residency program could be predicted from the number of weeks of pre-clerkship exposure (*p* = 0.01; *R* ^2^ = 0.36) but not from the number of clerkship weeks (*p* = 0.74).

**Conclusions:**

This study indicates that the duration of pre-clerkship exposure to psychiatry predicts the number of students selecting psychiatry as their first choice as a discipline. Thus, increasing the duration of pre-clerkship exposure may increase the enrollment of medical students into psychiatry.

Mental illness is a significant contributor to the global burden of disease and affects people in all communities across the world. The prevalence of mental illness is on the rise. According to a World Health Organization (WHO) 2012 declaration, mental illness has grown to become the leading cause of disability worldwide in terms of total years lost due to disability, leading the WHO to develop a comprehensive mental health action plan for the upcoming decade [1, 2].

Recruitment into psychiatry has been a persistent concern for decades in many countries, including the USA [3], Canada [4], Australia [5], and the United Kingdom [6, 7]. Declining recruitment has led to the question of whether or not psychiatrists are an endangered species [8]. Despite attempts to turn the tide, recruitment has not recovered from the downturn that became evident in the 1970s [6]. A study examining unmet needs for mental health professionals at the county level across the USA using data from the National Comorbidity Survey Replication (NCS-R) study found significant gaps in the availability of mental health care in the country. This study used a formula derived from the NCS-R data to provide estimates of prevalence of mental health issues in counties across the country and examined the time patients with mental health issues identified spending with prescriber and non-prescribers of mental health care on a yearly basis. These data were then extrapolated to “full-time equivalent (FTE)” need and cross matched with national mental health workforce patterns to identify areas of mental health need [9]. Overall results showed that over three quarters of the counties in the country had a severe shortage of mental health prescribers or non-prescribers, with nearly all counties (96 %) being found to have at least some prescriber shortage [10].

In 2010, the Canadian Medical Association estimated supply and demand statistics for psychiatry, specifically in Ontario. Assuming equilibrium (e.g., no major policy changes, migration shifts), a decrease is projected in the number of psychiatrists per population with the number of people per psychiatrist increasing from 7210 in 2010 to 8435 in 2030, a rise of about 15 % [11]. Additionally, a recent needs-based study from the Ontario Ministry of Health and Long-Term Care, in collaboration with the Ontario Medical Association, concluded that the province is already experiencing a shortage of psychiatrists, and this problem is projected to worsen significantly over the next 10 years [12]. Thus, expanding psychiatry training programs across Canada to meet patient demand is needed [11], and this need for expansion has been addressed with the number of training positions gradually increasing [13].

Unfortunately, in Canada, the percentage of applicants choosing psychiatry as a first choice has been gradually declining (Fig. 1). Although medical school enrollment and psychiatry residency positions are increasing, there are several indicators that psychiatry is “losing market share” in comparison with other medical specialties [14]. Consequently, Sargeant and colleagues [14] advocate for active measures to increase recruitment for psychiatry, rather than complacently waiting for the increased medical student enrolment to fill the growing available psychiatry residency positions passively.

**Fig. 1:**
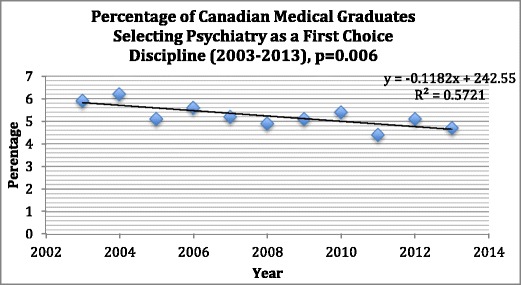
This figure shows a statistically significant declining interest in psychiatry as a first choice selection by medical students over the last 10 years

The decline in applicants choosing psychiatry as a first choice for medical specialty in Canada mirrors the experience of recruitment in the United Kingdom, with 15 % of psychiatry residency positions being unmatched in the first round in 2009 [7]. Consequently, re-entrant learners and second-choice applicants increasingly are filling training positions. In addition to these applicants, international applicants are also filling these positions increasingly, because they often are forced to enter the second round of the match. For instance, in the United Kingdom, the ratio of international to local graduates entering the psychiatry training program in 2009 was approximately 6:1 [15]. The challenges in recruitment into psychiatry in the USA are concerning as well. The percentage of medical students pursuing a psychiatry residency in the USA was 7–10 % in the 1940s but has lingered between 3 and 4 % since the 1990s; it remains a full percentage less than that of Canada [16, 17]. The factors leading to this difference remain unknown.

The increasing mental illness burden, expanding medical school class sizes, lengthy waiting times to see psychiatrists, and projected worsening of the shortages of psychiatrists in Canada provide an impetus for educators and directors of education to re-examine how we influence the distribution of physicians by specialty. By doing so, we can better meet the needs of our patients and our society at large. To this end, the Canadian Organization of Undergraduate Psychiatric Educators (COUPE) conducted a survey examining the characteristics of psychiatric training in the MD programs of the 17 medical schools in Canada. We explored the time to first exposure in psychiatry, the duration of pre-clerkship and clerkship, subspecialty exposure, availability of electives, the effect of longitudinal integrated clerkships, and provincial salaries and compared this information to the percentage of medical students choosing and then matching to psychiatry as their first choice. We speculated that these factors would be most influential in determining medical students’ career choice, and so we chose them for this exploratory study, as others have hypothesized that these factors influence medical student career choice, both for psychiatry and other specialties [18].

## Method

In 2012, COUPE conducted telephone interviews and sent e-mail questionnaires to the 17 medical schools across Canada as represented by the directors of education in psychiatry; all schools provided data. The questionnaires included data on the following: the duration of exposure to psychiatry in pre-clerkship and clerkship; the number of schools using case-based learning (CBL), team-based learning (TBL), problem-based learning (PBL), and *e*learning; opportunities for subspecialty experience (and whether or not they were mandatory, whether residents formally taught and were evaluated); opportunities for electives; on-call requirements; and average provincial salaries. The survey also asked whether residents are taught to teach and how they are evaluated. We asked about longitudinal integrated clerkships (LICs) and how students provide feedback to shape the curriculum. We obtained further data and relevant statistics from the Canadian Resident Matching Service [19]. We performed statistics using v12 STATA program (StataCorp, College Station, Texas, US) and Microsoft Excel and set significance at a *p* value of <0.05.

## Results

Table 1 summarizes selected data obtained from our survey. From the 17 medical schools in 2012, medical student enrollment ranged from 54 to 266 students (mean = 158 ± 16). Since 1968, medical school enrollment has gradually increased to 11,375 students nationwide [20]. We also explored, from a lifespan perspective, the opportunities for exposure to child psychiatry and geriatric psychiatry. Although the option for electives in geriatric psychiatry were similar across the Canadian medical schools at 94 %, the actual number of weeks that clerks rotated through these rotations in 2012 was less in geriatric (0.1 ± 0.3 weeks) than in child psychiatry (0.8 ± 0.9 weeks). This may have occurred because child psychiatry was frequently a mandatory part of the medical curriculum (53 % for child vs. 34 % in geriatrics), hence less opportunity for elective work in this area. Nationally, a similar number of schools offered electives in forensic psychiatry (94 %) but less so for addiction psychiatry (71 %). Most schools offered blended learning with didactic lectures (53 %), small group sessions (88 %), and electronic self-learning modules (65 %). The percentage of these students who ranked psychiatry as their first choice for residency specialty was 4.9 ± 0.6 % with final match results yielding similar numbers at 5.0 ± 0.6 %. Figure 1 shows the declining trend of students choosing psychiatry as their first choice in the first iteration of the Canadian Resident Matching Service (CaRMs) match from 2003 to 2013.

**Table 1:** Medical education and psychiatry experience of medical students in Canadian medical schools (2012)

	National averages
Number of students per medical school	158 ± 16
Duration of pre-clerkship (weeks)	2.8 ± 0.5
Duration of clerkship (weeks)	6.2 ± 0.3
Child psychiatry (weeks)	0.8 ± 0.9
Elective (16/17)	94 %
Mandatory (9/17)	53 %
Geriatric psychiatry (weeks)	0.1 ± 0.3
Elective (16/17)	94 %
Mandatory (6/17)	35 %
Forensic psychiatry (weeks)	0.6 ± 1.5
Elective option (16/17)	94 %
Addictions (weeks)	0.1 ± 0.5
Elective option (12/17)	71 %
On-call requirements (number per month)	7.2 ± 2.4
Didactic lectures	53 %
Small group teaching	88 %
Case-based learning (CBL) (% of schools)	100 %
Problem-based learning (PBL) (% of schools)	88 %
Team-based learning (TBL) (% of schools)	41 %
Self-learning modules (SLMs) (% of schools)	65 %
Simulation center use	41 %
ePortfolio/CanMEDS tracking	65 %
Longitudinal integrated clerkships (2/17)	12 %
First choice	4.9 ± 0.6 %
Final match	5.0 ± 0.6 %
Vacancies	43.4 ± 15.1 %

**Fig. 2:**
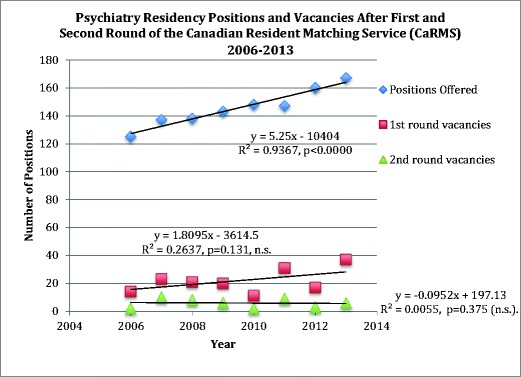
This figure shows that while the number of psychiatry residency positions being offered in Canada has increased significantly over time, the number of first round vacancies has seen only a slight increase over time. This suggests that while there are more medical school graduates choosing psychiatry to fill the increasing number of available positions, the increase in candidates is not keeping up with the number of available positions after the first round of the match. There is no significant increase in vacancies in the second round of the match, however, indicating that virtually all the positions are still being filled. It is likely these positions are being filled by re-entrant learners, students transferring from another specialty, or international medical graduates

**Fig. 3:**
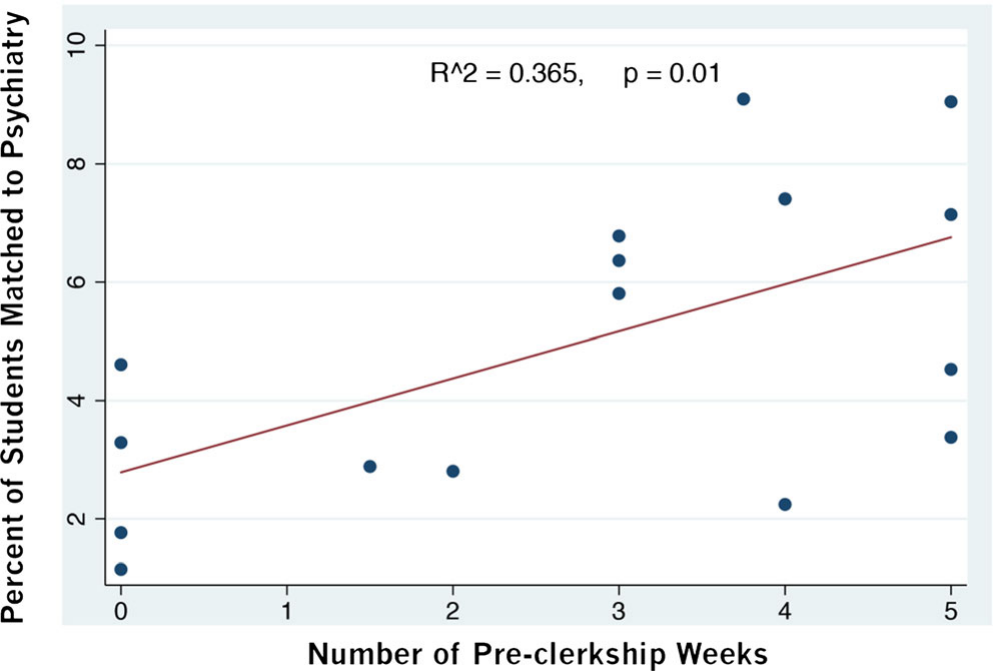
This figure illustrates that for medical schools in Canada, there is significant variability in the length of pre-clerkship weeks devoted to psychiatry education. This figure illustrates that in Canadian medical schools, a higher number of pre-clerkship weeks in psychiatry is strongly associated with a higher percentage of those medical students matching to psychiatry (*p* = 0.01)

In 2012, 10 out of 17 programs filled all psychiatry residency positions, while the remaining 7 programs had vacancy rates ranging from 5 to 100 % (mean = 43.4 ± 15.1 %). The available residency positions in each program ranged from 2 to 27 positions. In keeping with increased medical school enrollment, the number of residency positions has increased over time, psychiatry being no exception. Figure 2 demonstrates this increasingly linear trend. The number of vacancies present nationally after the first round is apparently increasing, as Fig. 2 further shows; however, the number of vacancies after the second iteration process does not appear to be increasing. Since the number of vacancies after the match is not increasing despite the increase in vacancies after the first round, this may suggest that psychiatry residency positions are being matched to either Canadian or international medical graduates who may not complete the respective program (i.e., they transfer into psychiatry from another specialty). Medical students were exposed to an average of 2.8 ± 0.5 pre-clerkship psychiatry weeks. Linear regression analysis showed that the percentage of graduating medical students entering a psychiatry residency program could be predicted by the number of weeks of pre-clerkship exposure (*p* = 0.01; *R* ^2^ = 0.36) (see Fig. 3).

**Fig. 4:**
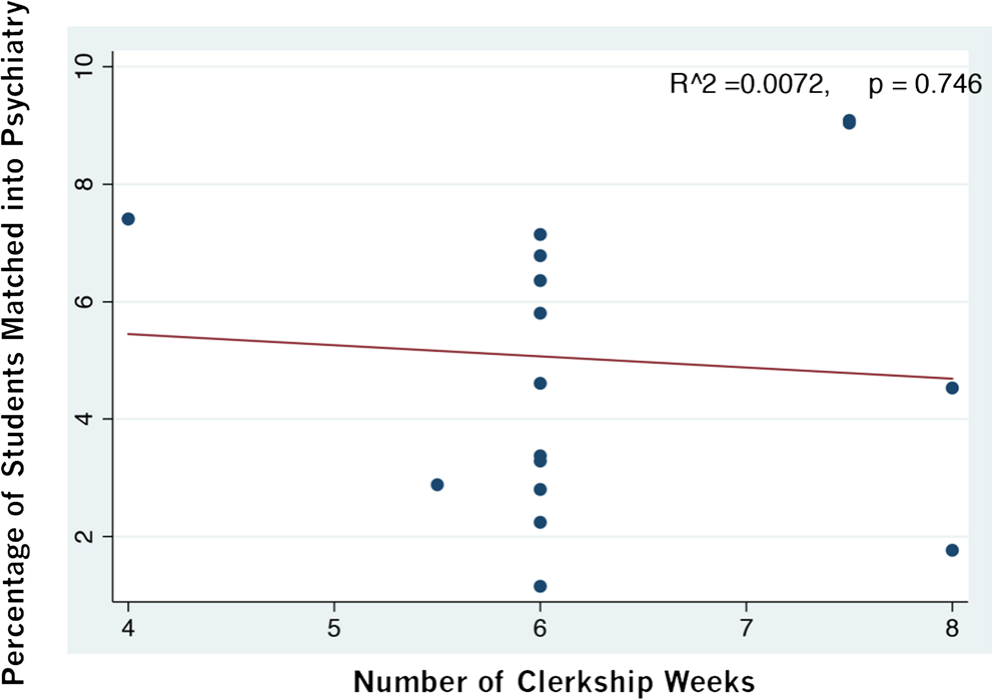
This figure illustrates that for medical schools in Canada, the number of clerkship weeks spent in psychiatry was not associated with medical students matching to psychiatry as a medical discipline. Most programs in Canada, however, have a similar number of clerkship weeks devoted to psychiatry, making it unlikely an association would emerge

Medical students spent an average of 6.2 ± 0.3 clerkship weeks across the country. We found no relationship for clerkship duration and percentage matching to psychiatry (*p* = 0.74; see Fig. 4). Provincial salaries similarly did not demonstrate a trend toward percentage matched to psychiatry, even when we made adjustments to exclude part-time physicians (i.e., those who made less than $60,000 per year) and to compare the relative provincial salary rather than gross national salary (data not shown). The experience of call frequency also did not have an effect on matching to psychiatry across Canada. Two of the 17 schools in Canada have longitudinal integrated clerkships, with one of the schools having sufficient exposure of students to provide data on percentage matching to psychiatry. The number of students applying to psychiatry from the integrated clerkship program is 3/38 (7.9 %) versus 67/397 from other clerkships (16.9 %). Although only about half as many applied from the integrated clerkship, this trend was not significant by the Fisher’s exact test (*p* = 0.11).

## Discussion

The factors leading to medical students choosing psychiatry are complex. In many countries, the recruitment problem into psychiatry is clear and evident and has been so for decades [8, 21–24]. Suggested solutions and promotional programs have not been effective overall, and uncertainty remains about future developments [5, 8]. Reasons for declining enrollment have been suggested, and they include the stigma of mental illness, difficulties of treating persons with mental illness, relatively lower income among specialists, lack of respect from medical peers, perceived weak scientific foundations, ineffective treatment, and uninspiring role models [5, 25–28]. Although many psychiatrists have advocated for increased exposure to psychiatry as a means of improving recruitment, a study in the 1990s of the medical schools in the USA found no clear correlation with the length of psychiatry clerkships and recruitment into psychiatry [29]. Recruitment into psychiatry at that time, however, was at its lowest point since 1929 [30]. It is intuitive that medical students’ exposure to psychiatry must have some effect on their interest in the field. Although quality psychiatry exposure typically trumps quantity, to achieve this “quality” exposure, students will require a sufficient quantity of exposure time. Spollen and colleagues [31], at the 2013 American Psychiatric Association meeting in San Francisco, presented survey data investigating psychiatry recruitment variables. Although they similarly found no association between length of clerkship and recruitment, they did see an association between schools that had relatively highly rated clerkships (perceived quality programs), level of stigma among non-psychiatrist faculty, and recruitment.

As stated previously, it is unclear why the USA has had more of a marked decline in recruitment into psychiatry than Canada and remains a full percentage point less per year [16, 17]. The average length of US medical school psychiatry clerkships has been gradually declining over the past 30 years, from 6.4 weeks in 1982, to 6 weeks in 1999, and to 5.5 weeks in 2010 [32]. Although psychiatry clerkships in Canada have also decreased over the last 30 years, they have not declined to the same extent. Overall, the duration of psychiatry clerkship exposure is decreasing in Canada, as is the interest in psychiatry nationally (Fig. 1). One medical school in Canada that went from 6 to 4 weeks clerkship in 2012 saw this cohort have none of its medical students matching into their local psychiatry program in 2013 [17]. As Fig. 4 demonstrates, we did not find a statistically significant association nationally of clerkship duration with percentage match to psychiatry at each of the schools. Although the absolute number of weeks of clerkship psychiatry exposure was not correlated with percentage match to psychiatry, several factors remain to be explored, such as the optimal structure for the psychiatric rotation itself, the perceived quality among medical students, and the depth of patient interaction. If there was a signal, it may be lost, because most of the schools have a 6-week duration of clerkship and an association, therefore, may be harder to demonstrate, because there was not much variance. Additional factors such as the stigma of psychiatry within medicine and the value of role modeling may also influence how psychiatry is viewed as a career choice. These factors, however, are more difficult to quantify and examine.

Hirsh and colleagues [33] recently noted that medical students who had longitudinal, integrated educational experiences “demonstrated richer perspectives on the course of illness, more insight into social determinants of illness and recovery, and increased commitment to patients” in comparison with students in a traditional training system. This contemporary system, that is, the longitudinal integrated clerkship (LIC), was conceived in hopes that more students may choose psychiatry as a career. They speculated that if students had more longitudinal exposure to these common conditions, they could develop more interest and have more opportunities to overcome the stigma associated with mental illness. To the contrary, we found that, in Canada, there was a close trend for students in LICs to be less likely to choose psychiatry in comparison with the regular stream, although the relationship did not reach statistical significance. This was an unexpected finding, because it is (almost) the opposite of what would be expected to occur on the basis of how the LIC experience is designed. There may be several reasons pertaining to how the longitudinal clerkships are set up, with rural experiences more likely to lead to students choosing family medicine: a more rurally based program may offer fewer opportunities for specialty immersion because most educators would be generalist psychiatrists or family medicine physicians.

To our knowledge, quantifying pre-clerkship exposure and correlating this exposure to interest in psychiatry has not been demonstrated. After linear regression analysis, we demonstrated that the length of pre-clerkship exposure predicted subsequent student interest in pursuing psychiatry residency training (first-choice discipline). This study has some limitations. These data were collected over a limited time, which may limit the generalizability of the findings. In general, pre-clerkship length, however, has nationally remained stable. Furthermore, the limited sample size limits the utility of linear regression in this study.

The most consistent findings to date on psychiatry recruitment variables suggest that early-career interest is the strongest predictive factor [4]. Increasing the duration of pre-clerkship psychiatry exposure without improving its quality may be of limited benefit to increasing early or later career interest, as the survey presented by Spollen and colleagues suggests [31]. Because quality of the clerkship experience is suggested to affect recruitment into psychiatry even though length of clerkship may not, it is reasonable to posit that if length of pre-clerkship does affect recruitment, then the quality of pre-clerkship may be even more important. Having more exposure to psychiatry in pre-clerkship early on may help challenge the ongoing stigma and misperceptions regarding psychiatry and patients with mental illness. The pre-clerkship educational experience could be well suited to support the biological underpinnings of mental illness and the success rates of psychiatric treatment in comparison with those of other medical specialties. This early pre-clerkship educational experience can be part of the basic science education that characterizes the exposure of students in their early formative years. Additionally, further research could examine the timing of pre-clerkship in the medical school curriculum as a potential additional factor in influencing psychiatry as a career choice. If students are exposed at the end of their pre-clerkship period, they may not have an opportunity to consider psychiatry as a career choice, when electives and other clerkship prerequisites have to be completed. Psychiatry, more than other specialties, is surrounded by stigma and stereotype, even at the medical school level. Exposure is the primary means by which the specialty can dispel these preconceived notions. Another issue that was not examined in these data but could be of future interest is whether the type of early exposure in the pre-clerkship period influences career choice, such as traditional didactic teaching versus clinical shadowing or small group learning experiences.

All medical schools should plan to increase exposure of students to areas of medicine that are underserviced. This includes both the curriculum and that which is hidden and embedded within. What medical students know of particular specialties often comes from the physician preceptors themselves. Encouraging psychiatrists to be mentors and having them be part of the introduction to clinical medicine and professionalism courses will likely be of benefit to recruitment. Other factors that could lead to increased initial exposure include student interest groups in pre-clerkship, faculty research projects, professional online groups, and access to complimentary psychiatry journals: most of these facets can be controlled at the medical school and psychiatric educator level.

Consistent with previous studies that suggest early-career interest as being predictive to recruitment, our study suggests that the duration of pre-clerkship exposure to psychiatry is associated with the number of students selecting psychiatry as their first choice as a discipline. The exposure may be a means of catalyzing early-career interest. Increasing the duration of pre-clerkship exposure may further improve the enrollment of medical students into psychiatry, especially if special efforts are made to enhance the quality of patient interaction. One of the Canadian medical schools with the highest match percentages to psychiatry consistently makes special efforts to enhance the quality of the pre-clerkship experience, including enhancing the quality of patient interaction.

For the sake of patients with mental illness and their families, it is imperative and a responsibility for leaders of medical education to continue to innovate and explore ways to increase the workforce of psychiatrists and other mental health professionals, to meet the increasing need for psychiatric services. Medical schools need to nurture interest in medical students early on in underserviced specialties like psychiatry, thereby investing in the future.
